# Transplant Trial Watch

**DOI:** 10.3389/ti.2022.10513

**Published:** 2022-05-16

**Authors:** John M. O’Callaghan

**Affiliations:** ^1^ University Hospitals Coventry and Warwickshire, Coventry, United Kingdom; ^2^ Centre for Evidence in Transplantation, Nuffield Department of Surgical Sciences, University of Oxford, Oxford, United Kingdom

**Keywords:** kidney transplantation, liver transplantation, everolimus, CMV infection, machine perfusion

To keep the transplantation community informed about recently published level 1 evidence in organ transplantation ESOT and the Centre for Evidence in Transplantation have developed the Transplant Trial Watch. The Transplant Trial Watch is a monthly overview of 10 new randomised controlled trials (RCTs) and systematic reviews. This page of Transplant International offers commentaries on methodological issues and clinical implications on two articles of particular interest from the CET Transplant Trial Watch monthly selection. For all high quality evidence in solid organ transplantation, visit the Transplant Library: www.transplantlibrary.com.

RANDOMISED CONTROLLED TRIAL 1 Incidence of Cytomegalovirus Infection in Seropositive Kidney Transplant Recipients Treated with Everolimus: a randomized, open-labelled, multicentre phase 4 trial.

*by Kaminski, H., et al. American Journal of Transplantation [record in progress].*

## Aims

The aim of this study was to investigate whether everolimus (EVR)-based immunosuppression leads to a decrease in the incidence of cytomegalovirus (CMV) DNAemia and disease.

## Interventions

Participants were randomised to receive either EVR or mycophenolic acid (MPA) combined with basiliximab, cyclosporin and steroids.

## Participants

186 CMV seropositive renal transplant recipients.

## Outcomes

The primary outcomes were CMV treatment, CMV DNAemia, patient death, graft loss and discontinuation of the study at 6 months following transplantation. The secondary outcomes were maximal viral load, the CMV treatment failure, proportion of patients with CMV disease, and the incidence of CMV mutations (UL97 or UL54) associated with a resistance to an anti-CMV therapy.

## Follow-up

12 months.

## CET Conclusion

This large, multicentre phase 4 RCT aimed to demonstrate whether everolimus-based immunosuppression is associated with a reduction in CMV viraemia following renal transplantation. Seropositive recipients were randomised to either everolimus or mycophenolic acid, in conjunction with cyclosporin and steroids. CMV prophylaxis was not used. The study used a composite primary endpoint of CMV treatment, graft loss, death and discontinuation, and showed that in intent-to-treat analysis there was a significant reduction in this endpoint with everolimus. This was driven mainly by a reduction in CMV DNAeamia in the everolimus arm. The study was stopped early due to findings from the ATHENA study that CsA and everolimus is associated with increased incidence of acute rejection–a finding that was not replicated in the present study. Nonetheless, as CMV infection rates were higher than anticipated the study has sufficient statistical power to demonstrate differences in outcome. Similar to previous studies, everolimus was poorly tolerated and benefit will be limited to those patients who can tolerate and maintain treatment. It is unclear how the present strategy compares to universal prophylaxis with more standard immunosuppression.

## Jadad Score

3.

## Data Analysis

Modified intention-to-treat analysis.

## Allocation Concealment

Yes.

## Trial Registration

ClinicalTrials.gov - NCT02328963.

## Funding Source

Industry funded.

RANDOMISED CONTROLLED TRIAL 2 Impact of Portable Normothermic Blood-Based Machine Perfusion on Outcomes of Liver Transplant: The OCS Liver PROTECT Randomized Clinical Trial.

*by Markmann, J. F., et al. JAMA Surgery 2022; 157 (3):189–198.*

## Aims

The aim of this study was to investigate liver transplant outcomes associated with portable normothermic machine perfusion preservation of livers obtained from deceased donors.

## Interventions

Participants were randomised to either the Organ Care System (OCS) group or ischemic cold storage (ICS) group.

## Participants

300 recipients receiving donor livers preserved using ICS or the OCS.

## Outcomes

The primary effectiveness outcome was the incidence of early allograft dysfunction (EAD). Secondary outcomes were extent of reperfusion syndrome, OCS Liver *ex vivo* assessment capability of donor allografts, incidence of ischemic biliary complications (IBCs) at 6 and 12 months, and overall patient survival posttransplant. The primary safety outcome was the number of severe adverse events related to the liver graft within 30 days following transplantation.

## Follow-up

1 year.

## CET Conclusions

This is an interesting and well-conducted, multicentre study in liver transplantation using a normothermic preservation machine (OCS). The study was adequately randomised and, understandably, clinicians could not be blinded to the group allocation, the comparator being standard cold storage on ice. However, good steps were taken to re-randomise patients if a first liver was subsequently not suitable for transplant. The donor population for inclusion was selected on the basis of at least one of the following criteria: 40 years of age or older; expected total cross-clamp/cold ischemic time of six or more hours; DCD donors if 55 years or younger; or macrosteatotic livers (≤40%). The primary endpoint was early allograft dysfunction (EAD) using the Olthoff definition. Mean perfusion time on the machine was 117 min, 152/155 preserved in this way were transplanted. However, there was a significantly higher proportion of DCD livers transplanted from the OCS group than the cold storage group (51% versus 26%). There were 298 patients included in the modified intention to treat analysis, which showed a significant decrease in EAD when the OCS machine was used compared to standard cold storage. Short term patient and graft survival was equivalent but ischaemic biliary lesions were significantly reduced with OCS by 6 and 12 months (2.6% versus 9.9%) and recipients experienced fewer incidences of severe reperfusion injury.

## Jadad Score

3.

## Data Analysis

Per protocol analysis.

## Allocation Concealment

Yes.

## Trial Registration

ClinicalTrials.gov - NCT02522871.

## Funding Source

Industry funded.

## Clinical Impact Summary

This is a well-conducted, multicentre study in liver transplantation using a normothermic preservation machine (The *OCS Liver* from *TransMedics, MA, United States*). The study took place over a period of approximately 3 years at 20 centres in the United States. The study targeted organs that had risk factors for early allograft dysfunction (EAD), such as older donor age, moderate steatosis, or anticipated long cold ischaemic time.

The study was adequately randomised and, understandably, clinicians could not be blinded to the group allocation. If a liver was found to be not suitable for transplantation, then the recipient was randomised a second time. This, to some extent, mediates any potential bias that might be introduced when clinicians could not be easily blinded to the preservation method.

Mean perfusion time on the machine was 117 min. The total preservation time for machine perfused livers was on average longer than the control group at 455 min compared to 339 min. Approximately 10% of livers randomised to OCS cross over to the other arm and were preserved with cold storage instead due to: accessory vessels, vascular reconstruction, or liver haematoma. However, the results from the intention to treat analysis were very similar to the per protocol analysis regardless, suggesting that there was no systematic bias introduced.

Reassuringly 98% of livers preserved on the machine were successfully transplanted; those not transplanted were not used following assessment on the machine, showing poor lactate clearance or fibrosis on biopsy. There was a significantly higher proportion of DCD livers transplanted from the machine perfusion group than the cold storage group (51% versus 26%).

The analysis showed a significant decrease in early allograft dysfunction (EAD, using the Olthoff definition) when the OCS machine was used. Short term patient and graft survival was equivalent but ischaemic biliary lesions were significantly reduced with OCS by 6 and 12 months (2.6% versus 9.9%).

This study shows the safety of this technology in liver preservation and how it can potentially give greater confidence to transplant livers following DCD or marginal DBD. Despite the greater proportion of DCD livers in the OCS machine group, and the longer overall preservation time, there was a lower incidence of severe reperfusion injury, EAD and ischaemic biliary lesions. This study adds weight to the improved preservation possible with normothermic machines, and the confidence in organ viability when using this platform.

